# Dietary intervention with Polygonatum sibiricum polysaccharides mitigates cadmium liver toxicity: a gut-liver axis perspective

**DOI:** 10.3389/fnut.2025.1583652

**Published:** 2025-07-16

**Authors:** Qiannan Di, Huimin Zhou, Huifang Chen, Xiaowei Wang, Xiao Huang

**Affiliations:** ^1^Public Health College, Shanghai University of Medicine and Health Sciences, Shanghai, China; ^2^Department of Clinical Nutrition, Beijing Jishuitan Hospital Guizhou Hospital, Guiyang, China; ^3^School of Public Health, Guizhou University of Traditional Chinese Medicine, Guiyang, China; ^4^Second Affiliated Hospital, Guizhou University of Traditional Chinese Medicine, Guiyang, China

**Keywords:** Cadmium, Polygonatum sibiricum polysaccharides, hepatotoxicity, gut microbiota, gut-liver axis

## Abstract

Cadmium (Cd) contamination in food chains poses a global health threat, necessitating safe and effective dietary interventions. While polysaccharides are emerging as detoxifying agents, the role of Polygonatum sibiricum polysaccharides (PSP) in Cd-induced liver injury remains unexplored. This study established a female rat model of cadmium (Cd)-induced liver toxicity with PSP supplementation (125 mg/kg/day) for 8 weeks. The effect of PSP on Cd-induced hepatotoxicity was evaluated through histopathological assessment, biochemical analysis, and measurements of Cd levels in the liver and kidneys. Metabolomics and gut microbiota analysis further explored the hepatoprotective mechanisms. Results demonstrated that PSP significantly reduced serum alanine aminotransferase (ALT) and aspartate aminotransferase (AST) levels in Cd-exposed rats, improved hepatic steatosis, and increased intestinal villi height. PSP decreased Cd accumulation in both the liver and kidney, enhanced intestinal barrier function, promoted the growth of beneficial bacteria (*Lactobacillus*), and modulated the production of short-chain fatty acids (SCFAs). These effects contribute to the alleviation of Cd-induced hepatic dysfunction and metabolic disorders, including pathways such as riboflavin metabolism, steroid hormone biosynthesis, nucleotide metabolism, purine metabolism, and 2-oxocarboxylic acid metabolism. In conclusion, PSP demonstrates potential as a functional dietary intervention for alleviating Cd-induced hepatotoxicity. This study advocates for PSP as a novel nutraceutical for mitigating dietary Cd toxicity.

## 1 Introduction

Accumulation of cadmium in produce is frequently regarded as a global food security challenge, given Cd’s persistent environmental mobility and propensity for biomagnification through trophic levels (1). In most cases, cereal crops serve as one of the major vectors for Cd entry into the human body through oral ingestion (2). Approximately, 10% of the global population is at risk for chronic low Cd exposure, and projections suggest a significant increase by 2040 (3, 4). The liver is a primary target for Cd accumulation and injury (3). Epidemiological and animal studies indicated that Cd exposure increased liver disease risk and promotes liver cancer (5, 6). As a result, growing health concerns have spurred increasing interest in biologically active compounds, particularly polysaccharides, due to their potential to mitigate Cd-induced toxicity (7).

Polygonatum sibiricum polysaccharides (PSP), a natural polysaccharide derived from the traditional medicinal and edible plant *Polygonatum sibiricum*, demonstrates potential dietary supplement for alleviating cadmium-associated liver damage in contaminated food. This aligns with the growing demand for nutraceuticals targeting environmental toxicant-induced health risks (8). Studies on mice indicated a protective effect against septic acute liver injury and non-alcoholic fatty liver disease, suggesting potential functional food for alleviating Cd-induced hepatotoxicity (9, 10). However, its specific role in ameliorating Cd-induced hepatotoxicity remains unclear.

Currently, a study by Iddrisu et al. (7) indicated that polysaccharides derived from *Polygonatum sibiricum* can form stable metal complexes, thereby alleviating oxidative stress, modulating immune responses, and against heavy metal toxicity. PSP preserves and enhances intestinal microbiota, stimulates *Lactobacillus faecium* proliferation and biofilm formation, regulates short-chain fatty acids (SCFAs) production and metabolism, and fosters beneficial microbiota (11–13). SCFAs are crucial for intestinal flora balance and mitigating liver dysfunction (14). Cd exposure disrupts gut microbiota homeostasis, exacerbating Cd accumulation and hindering excretion (15–18). Given PSP’s prebiotic properties and its ability to regulate intestinal tract probiotics, which can affect the occurrence and development of liver disease through the gut-liver axis, we hypothesize that PSP exerts protective effects against Cd-induced liver injury by improving the intestinal barrier and modifying the gut microbiota.

This study aims to evaluate PSP’s hepatoprotective potential in a Cd-induced liver injury rat model using histopathological examination, biochemical analysis, and metabolomics. Additionally, we explore the mechanisms by investigating PSP’s effects on gut microbiota, SCFAs, and intestinal barrier indicators.

## 2 Materials and methods

### 2.1 Animal experiments

Prior research indicates that females are more prone to Cd toxicity than males, owing to their higher absorption rates, and that the hepatotoxicity of Cd is more severe in females (19). Therefore, Female rat was selected for this experiment. Thirty four-week-old female Sprague-Dawley rats (65–75 g) from Guizhou University of Traditional Chinese Medicine (license SCXK (QIAN) 2021-0003) were acclimatized for a week in a specific pathogen-free (SPF) facility under controlled conditions: temperature 22 ± 2C°, relative humidity 40%–60%, 12/12 h light/dark cycle. Then randomly divided into three groups (*n* = 8): control (Con), Cd, and PSP. The study protocol was approved by the Animal Care and Use Committee of Guizhou University of Traditional Chinese Medicine (Approval No. 20230053), and all animals were treated humanely to minimize suffering. The Cd group received 50 mg/L CdCl_2_ in drinking water, reflecting human environmental exposure (20). The PSP group received 125 mg/kg/day PSP intragastrically for 8 weeks, confirmed safe in rats (21). According to the 2020 edition of the Chinese Pharmacopoeia, the recommended daily dosage of *Polygonatum sibiricum* ranges from 9 to 15 g. The 2025 edition of the Chinese Pharmacopoeia stipulates that the content of PSP, calculated as anhydrous glucose, in *Polygonatum sibiricum* herbal materials should not be less than 7.0%, equating to approximately 900 mg per day. For an adult weighing 65 kg, this translates to a dosage of about 12.5 mg/kg. In animal experiments, applying a 10-fold conversion factor yields a dosage of 125 mg/kg. The CdCl_2_ was obtained from Shanghai Yi En Chemical Reagent Co., Ltd. (Shanghai, China), and the PSP were sourced from Yuanye Biotechnology Co., Ltd., ensuring a purity level of no less than 70% (NO. S27804, shanghai, China). Food and water intake were monitored weekly. After 8 weeks, rats were euthanized, and serum, internal organs, colonic contents, liver, and small intestine tissue were collected, frozen with liquid nitrogen, and stored at −80C° for analysis.

### 2.2 Analysis of serum biochemistry

Serum concentrations of alanine aminotransferase (ALT) and aspartate aminotransferase (AST) were quantified to assess liver cell injury using a Siemens Advia 1800 automated analyzer, a device renowned for its precision and reliability, manufactured by Siemens Diagnostics in Berlin, Germany.

### 2.3 Measurement of Cd content

The concentrations of Cd in bone samples were determined using an inductively coupled plasma source mass spectrometer (iCAP RQ, ThermoFisher, United States). The detection was performed in accordance with standard methods outlined in the Chinese Standard GB 5009.268-2016 for Cd analysis. The main steps involved accurately weighing a suitable amount of sample (precise to 0.0001 g) into a glass container, adding 10 mL of a nitric acid-perchloric acid mixed solution, and digesting the mixture on an electric heating plate. If the digestion solution turned brown-black during the process, a small amount of additional mixed acid was appropriately added until white smoke was emitted, indicating complete digestion, and the solution became colorless and transparent or slightly yellow. After cooling, the solution was diluted with water to a total volume of 25 mL, mixed well, and set aside for further analysis. At the same time, a blank test was performed. The prepared test solution was then used to determine the Cd element content in the liver samples.

### 2.4 Rat liver and intestine histopathology histopathological examination

Hematoxylin and eosin (H&E) staining were conducted to assess tissue histological structure. Tissue sections underwent deparaffinization, rehydration through graded alcohols, staining with hematoxylin for 5 min, differentiation in acid alcohol, bluing in Scott’s tap water, rinsing with distilled water, staining with eosin for 3 min, dehydration through graded alcohols, clearing in xylene, and mounting with a coverslip using mounting medium.

### 2.5 Immunohistochemistry

Tissue sections were deparaffinized and rehydrated through a graded series of alcohol. Antigen retrieval was conducted by heating the sections in citrate buffer (pH 6.0) for 15 min. Endogenous peroxidase activity was quenched with 3% hydrogen peroxide for 10 min, followed by blocking with 5% bovine serum albumin for 30 min at room temperature. The sections were then incubated with primary antibodies overnight at 4C°, followed by incubation with appropriate secondary antibodies for 1 h at room temperature. Finally, the sections were visualized using 3,3′-diaminobenzidine (DAB) substrate, counterstained with hematoxylin, dehydrated, and mounted.

### 2.6 16S rRNA gene sequencing

Colonic microbiota DNA was extracted using the DNeasy PowerSoil kit (Qiagen, Germany) and confirmed by agarose gel electrophoresis. DNA concentration was measured using a NanoDrop spectrophotometer. PCR amplification targeted the V3-V4 regions of the bacterial 16S rRNA gene with modified universal primers containing sample-specific barcodes and Illumina sequencing adapters. Sequences were clustered into OTUs at 97% similarity, and representative sequences were identified. Comprehensive analysis included species-level identification, quantification, alpha diversity assessment, and community structure examination.

### 2.7 Detection of short-chain fatty acids in colonic contents

Stock solutions of six SCFAs (acetic, propionic, isobutyric, butyric, isovaleric, and valeric acids) and caproic acid were prepared at 100 mg/mL using water and ether. An internal standard (4-methylvaleric acid) solution at 375 μg/mL was also prepared. All chemicals were sourced from Sigma-Aldrich (Shanghai, China). A calibration curve with ten points (0.02–500 μg/mL) was constructed by mixing working solutions with phosphoric acid, internal standard, and ether. Stock solutions were stored at −20C°, and working solutions were freshly prepared. Samples were homogenized with water and glass beads, centrifuged, and processed by adding phosphoric acid, internal standard, and ether. After vortexing and centrifugation, the supernatant was analyzed by gas chromatography-mass spectrometry (GC-MS). The analysis was conducted using a Trace 1300 gas chromatograph from Thermo Fisher Scientific, United States. The system was equipped with a capillary column, specifically the Agilent HP-INNOWAX (30 m × 0.25 mm ID × 0.25 μm). Helium served as the carrier gas at a flow rate of 1 mL/min. Samples were introduced in split mode with a ratio of 10:1, using an injection volume of 1 μL at an injector temperature of 250C°. The ion source and MS transfer line temperatures were set at 300C° and 250C°, respectively. The column temperature was programmed with an initial setting of 90C°, then increased to 120C° at a rate of 10C°/min, followed by a rise to 150C° at 5C°/min, and finally held at 250C° for 2 min at a rate of 25C°/min. For metabolite detection, a mass spectrometric analysis was performed on an ISQ 7000 instrument from Thermo Fisher Scientific, United States, utilizing electron impact ionization. The Single Ion Monitoring (SIM) mode was employed with an electron energy of 70 eV.

### 2.8 Liver metabolomics analysis

The procedures for metabolite extraction, sample preparation, and subsequent liquid chromatography-tandem mass spectrometry (LC-MS/MS) analysis followed established methodologies (22, 23). Raw data were processed using Compound Discoverer 3.1.0, with normalization via probability quotient. One-way ANOVA compared metabolite abundance among three groups.

Differential metabolites that distinguished the groups were identified through the calculation of Variable Importance in Projection (VIP) scores, utilizing partial least squares discriminant analysis (PLS-DA) and Orthogonal Projection to Latent Structures Discriminant Analysis (OPLS-DA), facilitated by the SIMCA-P 14.0 version. For metabolites exhibiting a significant differential abundance, characterized by a fold change of at least 2 or no more than 0.5 (with a maximum threshold of 2), a *p*-value below 0.05, and a VIP score exceeding 1, Kyoto Encyclopedia of Genes and Genomes (KEGG) pathway enrichment analysis was conducted.

### 2.9 RNA extraction and quantitative reverse transcription polymerase chain reaction (RT-qPCR)

Total RNA extraction from tissue was performed using the RNA isolator Total RNA Extraction Reagent (Nanjing Vazyme Biotech Co., Ltd., catalog number R401-01), with the specific procedural steps adhered to as outlined in the product manual (24). Total RNA was reverse transcribed into cDNA using HiScript IV All-in-One Ultra RT SuperMix (Vazyme, Nanjing). qPCR assessed mRNA expression levels of occludin and claudin, normalized to beta-actin, using Taq Pro Universal SYBR qPCR Master Mix. Primer sequences were: occludin (forward: 5′-CTTTTGAGAGTCCACCT-3′; reverse: 5′-GTCTTCCGGGTAAAAAGA-3′), claudin (forward: 5′-GGGGACAACATCGTGACCG-3′; reverse: 5′-AGGAGTCGAAGACTTTGCAC-3′), and beta-actin (forward: 5′-GGAGATTACTGCCCTGGCTC-3′; reverse: 5′-GACTCATCGTACTCCTGCTT-3′).

### 2.10 Statistical analysis

SPSS 26.0 software was used for statistical data analysis. The measurement data were expressed as mean ± standard deviation. One-way ANOVA was used to compare multiple groups, and the LSD test was employed for pairwise comparisons. A *p*-value of <0.05 was considered statistically significant.

## 3 Results

### 3.1 PSP ameliorated Cd-induced hepatotoxicity

**FIGURE 1:**
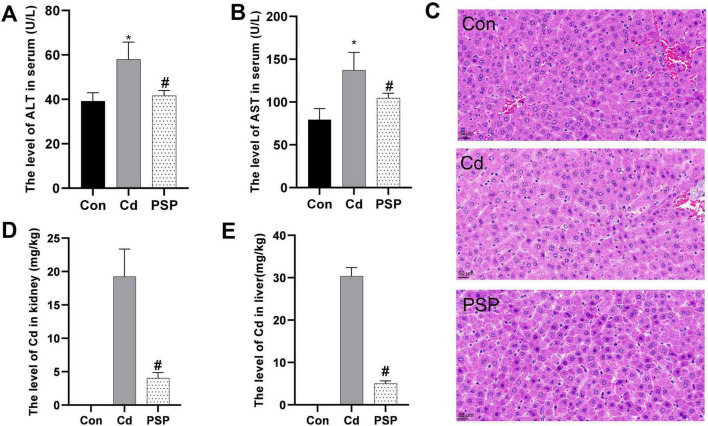
Polygonatum sibiricum polysaccharides (PSP) ameliorated Cd-induced hepatotoxicity and reduced Cd accumulation levels in the liver and kidney. **(A)** The level of AST in serum (U/L). **(B)** The level of ALT in serum (U/L). **(C)** Representative image of H&E staining from rat liver sections; **(D)** the level of Cd in kidney (mg/kg). **(E)** The level of Cd in liver (mg/kg). Data are shown as mean ± SEM, *n* = 8, significant differences to Con group are denoted by, *p < 0.05; significant differences compared to Cd group are denoted by, #*p* < 0.05.

Compared to the control group, serum ALT and AST levels were significantly elevated in the Cd group but significantly reduced in the PSP group (Figures 1A, B). Histopathological analysis showed that the hepatic lobule structure in the Con group was intact, with clear contours and uniformly sized, morphologically normal hepatocytes. In contrast, the Cd group exhibited preserved hepatic lobule structure, but hepatocytes contained variably sized vacuoles with distinct edges, indicating cellular damage. However, in the PSP group a marked reduction in the number of intracellular vacuoles was observed. Based on the hepatocyte ballooning score of the Brunt scoring system, the control group, Cd group, and PSP group had scores of 1, 2, and 1, respectively. These findings suggest that PSP may exert a protective effect against Cd-induced liver damage by promoting hepatocyte regeneration and reducing the formation of intracellular vacuoles (Figure 1C). Additionally, PSP intervention significantly decreased Cd levels in both liver and kidneys (Figures 1D, E).

### 3.2 Alterations in liver metabolomic profile

**FIGURE 2:**
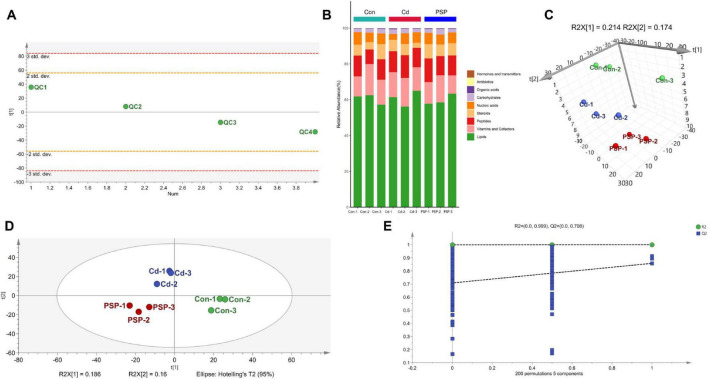
Alterations in liver metabolomic profile. **(A)** PCA analysis on the QC Samples. **(B)** The stacked column chart of the percentage of metabolites with a biological role. **(C)** 3D score plot of PCA model. **(D)** Score plot of PLS-DA model. **(E)** 200-iteration of permutation test (PLS-DA).

During detection, QC samples remained stable within 2 standard deviations (Figure 2A), ensuring data reliability. A total of 1865 metabolites, predominantly lipids, were detected (Figure 2B). PCA analysis distinguished the three groups (Figure 2C), and PLS-DA score plots confirmed clear separation (Figure 2D), with 200 permutation tests validating model reliability (Figure 2E).

**FIGURE 3:**
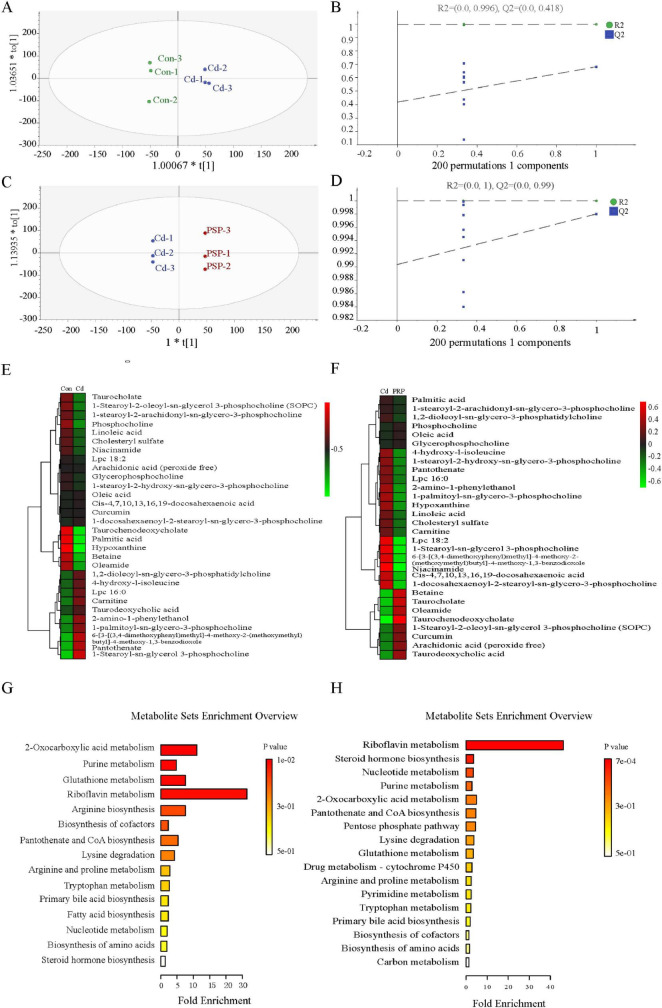
The results of the metabolomics analysis were based on the OPLS-DA model analysis. **(A)** The score plot of Con *vs.* Cd;**(B)** 200-iteration of permutation test (Con *vs.* Cd); **(C)** the score plot of Cd *vs.* PSP; **(D)** 200-iteration of permutation test (Cd *vs.* PSP); **(E)** the heatmap of Con *vs.* Cd differential metabolites; **(F)** the heatmap of Cd *vs.* PSP differential metabolites; **(G)** the metabolic pathways with significantly enriched differential metabolites in the Con *vs.* Cd; **(H)** the metabolic pathways with significantly enriched differential metabolites in the Cd *vs.* PSP.

Orthogonal Projection to Latent Structures Discriminant Analysis further delineated differential metabolites, showing significant separation between Con *vs.* Cd groups (R^2^X = 0.489, R^2^Y = 0.998, Q^2^ = 0.305) and Cd *vs.* PSP groups (R^2^X = 0.818, R^2^Y = 1.0, Q^2^ = 0.998) (Figures 3A, C), with 200 permutation tests confirming reliability (Figures 3B, D). Differential metabolites were detailed in Figures 3E, F. Compared to the control group, the proportion of lipid metabolites in the liver was significantly elevated in the Cd group but significantly reduced in the PSP group (Figure 3G). The metabolic pathways that were significantly enriched in the comparison between Cd *vs.* PSP include Riboflavin Metabolism, Steroid Hormone Biosynthesis, Nucleotide Metabolism, Purine Metabolism, and 2-Oxocarboxylic Acid Metabolism (Figure 3H).

### 3.3 PSP ameliorates intestinal barrier dysfunction under Cd exposure

**FIGURE 4:**
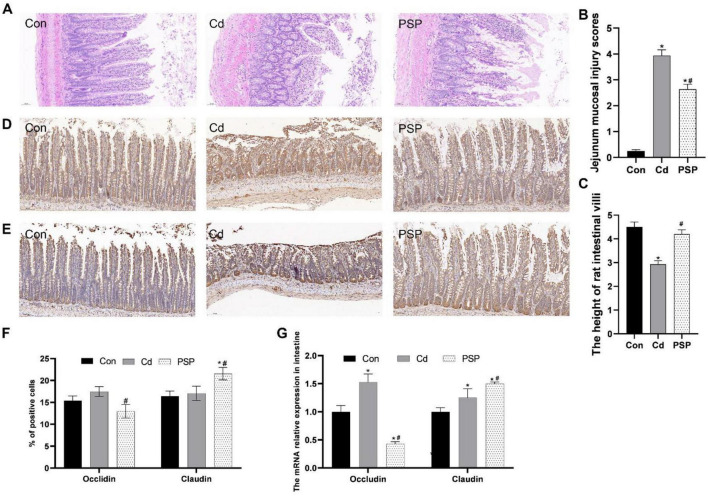
Polygonatum sibiricum polysaccharides (PSP) repaired the gut barrier in Cd-induced small intestine rat. **(A)** Representative images of H&E staining of small intestine tissue. **(B)** Jejunum mucosal injury scores based on H&E-stained histological sections. **(C)** Morphometric measurement of intestinal villus height in H&E-stained sections. **(D)** Representative images of immunohistochemical results of Occludin in small intestine tissue; **(E)** representative images of immunohistochemical results of Claudin in small intestine tissue; **(F)** quantitative analysis results of Occludin and Claudin according to immumohistochemical staining, *n* = 3. **(G)** Relative expression levels of Occludin and Claudin mRNA in small intestine tissue, *n* = 3. Data are shown as mean ± SEM, significant differences to Con group are denoted by, *p < 0.05; significant differences compared to Cd group are denoted by, #*p* < 0.05.

As shown in Figure 4A, the small intestinal tissues from the Con group were characterized by distinct stratification, with the mucosal epithelial cells being orderly aligned. The cell nuclei were clearly discernible, devoid of any notable atypia or evidence of mitotic activity. The villi were expectedly distinct, exhibiting a typical appearance. The crypts remained undilated and unaltered, with no significant infiltration of inflammatory cells observed. Conversely, in the Cd-exposed group, the architecture of the small intestinal villi was compromised, marked by detachment of the epithelial cells and aberrant crypt structures, and the presence of inflammatory cell infiltration. The jejunal mucosal injury scores were significantly increased in the Cd group compared with the Con group, while PSP treatment markedly attenuated this pathological alteration (Figure 4B). Cd exposure significantly reduced intestinal villus height versus the control group, which was effectively ameliorated by PSP intervention (Figure 4C). Nevertheless, the detrimental effects of Cd exposure on the small intestinal tissues were mitigated by the administration of PSP. Immunohistochemistry results showed (Figures 4D, E, F) that, compared with the control group, the expression level of Occludin in the Cd group was increased but not significantly different statistically. Compared with the Cd group, the expression level of Occludin in the PSP group was significantly decreased. Compared with the control group, the expression level of Claudin in the Cd group tended to increase but there was no significant difference. Compared with both the Con group and Cd group, the expression level of Claudin in the PSP group was significantly increased. As shown in Figure 4G, compared with the control group, the mRNA expression levels of Occludin and Claudin were all significantly increased. However, in the PSP group, the mRNA expression level of Occludin was significantly decreased, while the expression level of Claudin was significantly increased.

### 3.4 Alterations in gut microbiota

**FIGURE 5:**
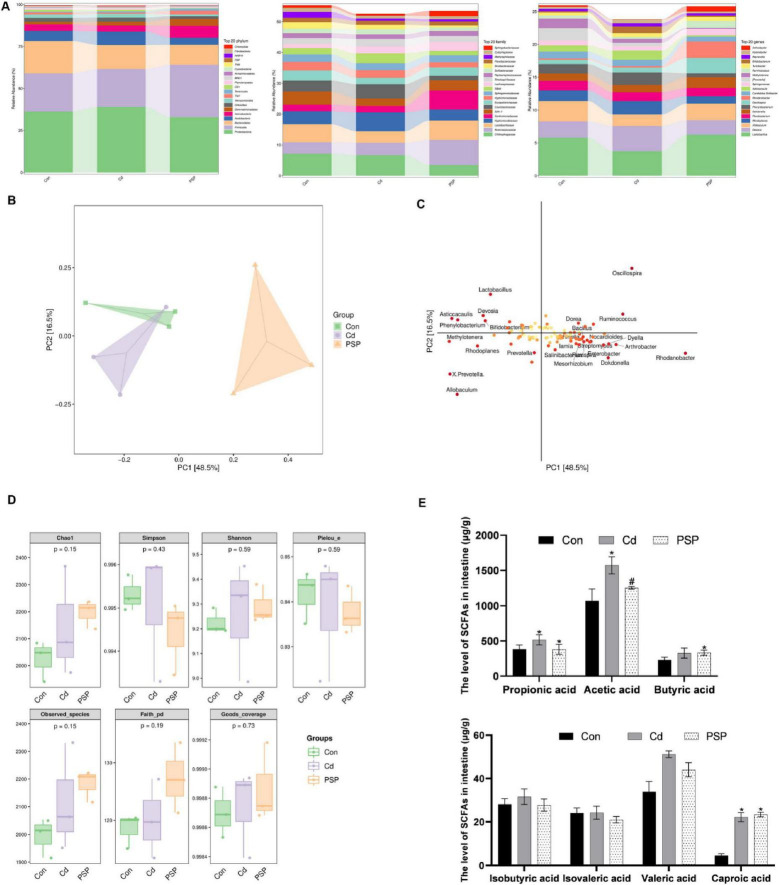
Alterations in gut microbiota in cecal contents. **(A)** Column chart of species composition at the phylum, family, and genus levels. **(B)** Score plot of PLS-DA analysis. **(C)** Loading plot of PLS-DA analysis. **(D)** Alpha-diversity-related indices. **(E)** Levels of short-chain fatty acids in intestinal, *n* = 3. Data are shown as mean ± SEM, significant differences to Con group are denoted by, *p < 0.05; significant differences compared to Cd group are denoted by, #*p* < 0.05.

To thoroughly explore the changes in intestinal microbiota, the intestinal contents of the colon were detected. As shown in Figure 5A, the distributions of intestinal microbiota at the phylum, family, and genus levels exhibited differences. At the phylum level, the top five in the Con, Cd, and PSP groups were respectively: *Proteobacteria* (36.30%, 39.07%, 32.93%), *Firmicutes* (22.68%, 22.52%, 31.23%), *Bacteroidetes* (19.24%, 14.35%, 11.90%), *Acidobacteria* (6.08%, 7.91%, 4.17%), *Actinobacteria* (3.77%, 3.70%, 7.10%). At the family level, the top five in the Con, Cd, and PSP groups were respectively: *Chitinophagaceae* (7.17%, 6.71%, 3.51%), *Ruminococcaceae* (3.72%, 4.01%, 8.17%), *Lactobacillaceae* (5.82%, 3.74%, 6.26%), *Hyphomicrobiaceae* (4.21%, 6.09%, 3.55%), *Xanthomonadaceae* (2.12%, 2.13%, 6.17%).

The β-Diversity analysis revealed that the populations among the three groups exhibited a separation trend (Figure 5B). The loading plot indicates that *Lactobacillus, Devosia, Asticcacaulis, Phenylobacterium, Bifidobacterium, Oscillospira, Dorea, Ruminococcus, Bacillus, Methylotenera, Rhodoplanes, Prevotella, X. Prevotella, Allobaculum, Dyella, Nocardioides, Lamia, Arthrobacter, Salinibacter, Rhodanobacter, Mesorhizobium, Dokdonella, Flexispira*, and *Enterobacter* were significantly different bacteria (Figure 5C). There were no significant differences in the α-diversity-related indices (Figure 5D).

As shown in Table 1, the correlation coefficients (*r*) of all short-chain fatty acid standard curves exceeded 0.995, with linear ranges covering the concentrations required for the experiment. As shown in Figure 5E, compared with the control group, the levels of Propionic acid, Acetic acid, Butyric acid, Isobutyric acid, Isovaleric acid, Valeric acid, and Caproic acid in the intestinal contents of the Cd group were all increased, among which Propionic acid, Acetic acid, Valeric acid, and Caproic acid were significantly increased. PSP intervention could reduce the increase in the levels of Propionic acid, Acetic acid, Butyric acid, Isobutyric acid and Valeric acid caused by Cd exposure. Among them, the level of Acetic acid in the PSP group was significantly decreased compared with that in the Cd group.

**TABLE 1:** Short-chain fatty acid standard curve.

Metabolism_name	Calibration curve	*r*	Linear range	LOQ
Isobutyric acid	*y* = 0.01061x−0.0001655	0.995	0.02–500.0	0.02
Propionic acid	*y* = 0.006504x + 0.0002888	0.996	0.02–500.0	0.02
Acetic acid	*y* = 0.004264x + 0.01188	0.995	0.02–500.0	0.02
Butyric acid	*y* = 0.02312x + 0.0004185	0.996	0.02–500.0	0.02
Valeric acid	*y* = 0.02815x + 0.0001022	0.996	0.02–250.0	0.02
Caproic acid	*y* = 0.1024x + 0.002874	0.995	0.02–250.0	0.02

### 3.5 Prediction analysis of 16s-based MetaCyc function in PSP intervention

**FIGURE 6:**
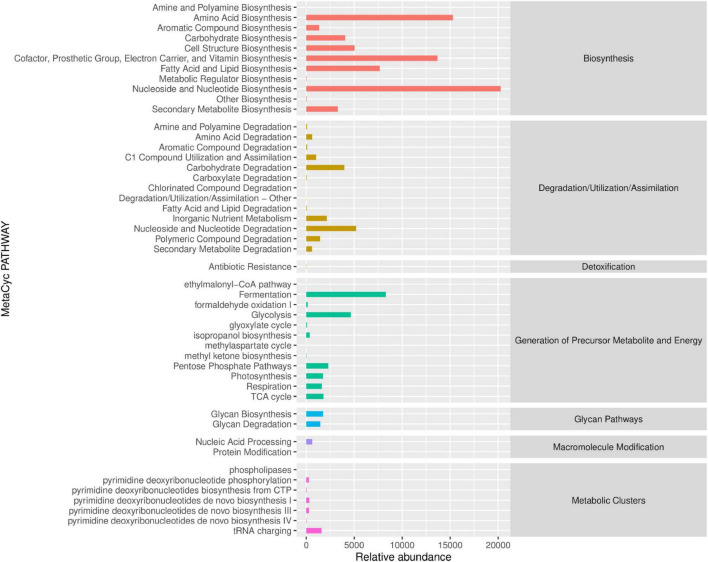
Prediction analysis of 16S rRNA-based MetaCyc functional pathways following PSP treatment.

The MetaCyc pathway abundance map clearly illustrates the activity levels of specific pathways following PSP intervention (Figure 6). The results indicate that the primary metabolic pathways significantly altered by PSP intervention, in descending order, were Biosynthesis, Generation of Precursor Metabolite and Energy, Degradation/Utilization/Assimilation, Metabolic Clusters, and Macromolecule Modification. Within the Biosynthesis metabolic pathway, nucleoside and nucleotide biosynthesis exhibit the highest relative abundance, followed by amino acid biosynthesis, as well as the biosynthesis of cofactors, prosthetic groups, electron carriers, and vitamins, and Fatty Acid and Lipid Biosynthesis. In the Generation of Precursor Metabolite and Energy metabolic pathway, fermentation shows the highest relative abundance, followed by glycolysis. Within the Degradation/Utilization/Assimilation metabolic pathway, Nucleoside and Nucleotide Degradation has the highest relative abundance, followed by Carbohydrate Degradation. In the Metabolic Clusters metabolic pathway, tRNA charging displays the highest relative abundance.

These metabolic changes imply that PSP may improve liver function by enhancing cellular repair and regeneration, supporting energy metabolism, and facilitating the degradation of harmful substances. Specifically, the enhancement of nucleoside and nucleotide biosynthesis, along with the support for protein synthesis and energy production, suggests that PSP may help mitigate the liver damage caused by cadmium by promoting cell repair and maintaining cellular integrity.

## 4 Discussion

Dietary supplements are a strategy for mitigating Cd toxicity. Our study investigated PSP′s the potential benefits in a rat model of Cd-induced liver injury. PSP significantly reduced liver function markers (ALT, AST). Histopathological analysis corroborated PSP′s protective effect, showing improved hepatocyte arrangement, reduced microvacuolation, and mitigated fatty degeneration. Themetabolomics analysis showed a significant elevation of liver lipid metabolites in the Cd group compared to the control, which was markedly reduced in the PSP group, suggesting PSP′s protective effect on liver lipid metabolism. This finding aligns with recent studies that have implicated the imbalance of lipid homeostasis as a significant contributing factor in Cd-induced liver disease (6, 25, 26). To gain further insights into the mechanisms underlying PSP′s protective effects, we conducted a metabolomic KEGG pathway analysis. The analysis identified disruptions in several key metabolic pathways, including riboflavin metabolism, steroid hormone biosynthesis, nucleotide metabolism, purine metabolism, and 2-oxocarboxylic acid metabolism, suggesting PSP′s interference in these pathways. This interference may influence liver lipid metabolism and contribute to protection against Cd-induced liver injury. To sum up, the collective evidence from biochemical, histological, and liver metabolite analysis illustrates the efficacy of PSP in attenuating Cd-induced liver injury, supporting its potential as a dietary supplement for mitigating Cd toxicity.

Polygonatum sibiricum polysaccharides intervention effectively attenuated Cd-induced hepatotoxicity by restoring gut microbiota balance (e.g., enriching *Lactobacillus* spp.) and enhancing intestinal barrier integrity, which collectively reduced systemic Cd absorption and hepatic oxidative stress. These findings highlight PSP′s dual role as a prebiotic and detoxifying agent, positioning it as a promising dietary strategy for populations exposed to Cd-contaminated diets.

The intestinal barrier serves as a critical defense mechanism, for preventing harmful substances like Cd from penetrating into the circulatory system (27–29). Studies have shown that Cd exposure disrupts the intestinal paracellular barrier by increasing paracellular permeability, which is caused by the disruption of junctional function and structure. This, in turn, leads to increased absorption and accumulation of Cd in the body (27–29). Our study found that the PSP group had significantly lower Cd concentrations in the kidneys and liver compared to the Cd group. These results suggested that PSP intervention effectively decreased the accumulation of Cd. The histopathological examination of the ileum indicated that Cd exposure caused significant damage, characterized by epithelial cell shedding, a reduction in goblet cells, and substantial infiltration of inflammatory cells. However, after PSP intervention, we observed a mitigation of this damage, with preserved intact villi, a well-arranged intestinal epithelium, and an increased number of goblet cells. Furthermore, RT-qPCR and immunohistochemistry analysis revealed alterations of Occludin and Claudin in the PSP group compared to the Cd group, indicating a positive effect of PSP on intestinal barrier repair (30). This study suggests that PSP may facilitate the reduction of hepatic Cd levels through multiple pathways, including chelation, enhanced excretion, or reduced absorption. Prior research has corroborated the efficacy of polysaccharides in promoting fecal Cd excretion. For instance, purslane polysaccharide (PP) can significantly decrease Cd levels in colon tissues and facilitate Cd excretion through feces. Besides this, PP achieves the effect (of reducing Cd levels and promoting its excretion) by inhibiting the FXR-FGF15 axis in the intestine, thereby blocking the enterohepatic circulation of cadmium and further reducing its reabsorption within the body. Additionally, polysaccharides can indirectly enhance Cd excretion by modulating the structure of the gut microbiota. For example, PP increases the abundance of beneficial bacteria such as *Lactobacillus*, while reducing the presence of pathogenic bacteria like *Lachnospiraceae_NK4A136_group*. The SCFAs such as butyrate, produced by probiotics during the fermentation of polysaccharides, possess anti-inflammatory properties and can strengthen intestinal barrier function, thus reducing Cd absorption. Our observed hepatic Cd reduction aligns with this chelation-excretion paradigm, though future isotopic tracing (^111^Cd) will precisely partition absorption/excretion ratios. Collectively, these results indicate that PSP′s ability to alleviate the hepatotoxic effects of Cd is closely linked to its role in restoring the intestinal barrier, thereby reducing Cd absorption and accumulation.

The gut and liver interact in a variety of ways (31). The intestinal flora can regulate nutrient absorption, host immunity, hormone, drug, and toxin metabolism (31). This flora is, in turn, regulated by bile acids, IgA, and antimicrobial peptides secreted by the liver. Currently, modulation of the gut microbiota and/or their metabolites represents a potential therapeutic approach for the treatment of liver diseases (31). To investigate PSP′s hepatoprotective mechanism against Cd exposure, we examined the intestinal flora and SCFAs in rat colons. Heatmaps at the genus level demonstrated a reduction in *Lactobacillus* relative abundance after Cd exposure, which notably rebounded with PSP intervention. LEfSe analysis further corroborated these variations, indicating that Cd exposure disrupts gut microbiota and PSP can alleviate this dysbiosis to a certain extent (32). PSP contains various sugars, including fructose, rhamnose, glucose, mannose, galactose, xylose, and galacturonic acid (33). Among these components, PSP-W-1, a polysaccharide containing only galactose, has been found to significantly increase the diversity of intestinal flora and promote the growth of beneficial bacteria, such as *norank_f_Muribaculaceae*, *Lactobacillus*, and *norank_f_noranko_Clostridia_UCG-014*, while inhibiting the growth of harmful bacteria (33). In this study, PSP alleviates the imbalance of intestinal flora caused by Cd exposure, promotes the growth of beneficial bacteria (*Lactobacillus*), and inhibits the reproduction of potentially harmful bacteria (*Blautia, Variovorax, Chitinophaga, Asticcacaulis, Bacteroides)*. These changes may ameliorate intestinal metabolic disorders caused by Cd exposure and improve the immune and inflammatory responses triggered by the same, subsequently alleviating liver injury through the gut-liver axis.

Short-chain fatty acids are crucial for maintaining the integrity of the intestinal barrier and serve as an energy source for cells in the intestinal environment (34). In our study, we observed a significant increase in SCFAs, including acetic acid, propionic acid, and butyric acid, in the PSP group compared to Cd-exposed rats. This increase is likely due to PSP′s ability to enhance the expression of genes related to acetic acid and propionic acid metabolism in *L. faecis*, as previously reported (12). Furthermore, PSP has been shown to decrease intestinal pH, enhance SCFA generation, and increase the relative abundance of beneficial bacteria such as *Bifidobacterium, Lactobacillus*, and *Bacteroides* (35). Consistent with this, we found an increase in *Lactobacillus* after PSP intervention. *Lactobacillus* is a promising probiotic with antimicrobial activity, anti-inflammatory effects, and the capacity to maintain gut microbiota balance (36). Importantly, *Lactobacillus* is also a significant source of SCFAs (36, 37). Considering the established roles of SCFAs in modulating inflammatory reactions and alleviating liver damage, it was speculated that the protective effect of PSP against Cd-induced liver injury might be related to its ability to regulate SCFAs. Specifically, it was found that PSP increased the abundance of beneficial bacteria such as Lactobacillus, resulting in elevated production of SCFAs. Ultimately, this increase in SCFAs was postulated to potentially mediate PSP′s beneficial effects through the gut-liver axis, thereby alleviating Cd-induced liver damage. While this pilot study provides the first evidence of PSP′s detoxifying effects, further validation in larger cohorts and dose-ranging trials is warranted to establish its optimal dietary application. Nevertheless, the integration of multi-omics data offers actionable insights for designing targeted nutritional interventions against Cd toxicity.

This study has several limitations. Firstly, the relationship between specific microbial taxa and liver health outcomes remains purely correlational, necessitating further verification of causality within the Gut-Liver Axis. Future investigations will validate the pivotal role of the microbiota through fecal microbiota transplantation (FMT). To comprehensively analyze functional pathways, these studies will employ a multi-omics integration strategy (such as 16S rRNA sequencing combined with metagenomics and metabolomics) for verification. Secondly, only a single dose level of PSP was used in this study. Subsequent research will incorporate multiple doses to determine the optimal thresholds for efficacy and safety. Finally, the use of a female rat model, which ignores gender-specific differences in hormone regulation, immune response, and repair capacity, may lead to an overestimation or underestimation of the actual protective effect observed in male individuals.

## Conclusion

In conclusion, the research has demonstrated that PSP intervention effectively ameliorates Cd-induced hepatic injury in a rat model. PSP alleviates Cd-induced hepatotoxicity through synergistic effects involving multiple targets and pathways. The main protective mechanisms include the direct reduction of Cd accumulation and the improvement of hepatic metabolic pathways. PSP has been shown to rectify metabolic disruptions within the liver caused by Cd, with a particular focus on pathways such as riboflavin metabolism, steroid hormone synthesis, nucleotide metabolism, purine metabolism, and 2-oxocarboxylic acid metabolism. Concurrently, it stimulates the proliferation of beneficial gut bacteria, promotes the production of SCFAs, and repairs the intestinal barrier, all of which collectively contribute to the mitigation of Cd-induced hepatotoxicity. PSP, as a therapeutic agent, shows promise in ameliorating Cd-induced hepatic damage, suggesting its potential as a candidate for development as a functional foodstuff for the prevention and treatment of Cd-associated pathologies. This study lays a scientific foundation for precise nutritional interventions in populations at high risk of cadmium exposure, such as residents in contaminated areas.

## Data Availability

The original contributions presented in this study are included in this article/supplementary material, further inquiries can be directed to the corresponding author.
